# Soft Liver Phantom with a Hollow Biliary System

**DOI:** 10.1007/s10439-021-02726-x

**Published:** 2021-02-16

**Authors:** Xiangzhou Tan, Dandan Li, Moonkwang Jeong, Tingting Yu, Zhichao Ma, Saif Afat, Karl-Enrst Grund, Tian Qiu

**Affiliations:** 1 Cyber Valley Research Group, Institute of Physical Chemistry, University of Stuttgart, Pfaffenwaldring 55, 70569 Stuttgart, Germany; 2 Department of General, Visceral and Transplant Surgery, University Hospital Tuebingen, 72072 Tuebingen, Germany; 3 Micro Nano and Molecular Systems Lab, Max Planck Institute for Intelligent Systems, Heisenbergstr. 3, 70569 Stuttgart, Germany; 4 Department of Interventional and Diagnostic Radiology, University Hospital Tuebingen, 72072 Tuebingen, Germany; 5 Department of General Surgery, Xiangya Hospital Central South University, Changsha, 410008 China

**Keywords:** Liver model, Biliary interventions, Organ phantom, 3D printing, Interventional surgical training, Centesis, Ultrasound imaging, Electrical sensing

## Abstract

Hepatobiliary interventions are regarded as difficult minimally-invasive procedures that require experience and skills of physicians. To facilitate the surgical training, we develop a soft, high-fidelity and durable liver phantom with detailed morphology. The phantom is anatomically accurate and feasible for the multi-modality medical imaging, including computer tomography (CT), ultrasound, and endoscopy. The CT results show that the phantom resembles the detailed anatomy of real livers including the biliary ducts, with a spatial root mean square error (RMSE) of 1.7 ± 0.7 mm and 0.9 ± 0.2 mm for the biliary duct and the liver outer shape, respectively. The sonographic signals and the endoscopic appearance highly mimic those of the real organ. An electric sensing system was developed for the real-time quantitative tracking of the transhepatic puncturing needle. The fabrication method herein is accurate and reproducible, and the needle tracking system offers a robust and general approach to evaluate the centesis outcome.

**Supplementary Information:**

The online version contains supplementary material available at 10.1007/s10439-021-02726-x.

## Introduction

Simulation-based training has been recognized as an effective and safe approach to improve the clinical capabilities of medical professionals. Many studies show that surgical simulators are able to improve the early learning curve of novices, offer a dedicated learning environment and avoid patients’ discomfort or even possible complications.^28^ Organ models, which resemble the physical properties of human organs, are significant parts of the surgical simulators.

Surgical simulations are performed on various simulators including live animal models, *ex vivo* models, virtual reality (VR) models and mechanical simulators.^26^ Although living animal models and *ex vivo* models use real biological tissues, they exhibit severe disadvantages such as the inconsistency to human anatomic structures, non-reusable, non-standard, high-cost, requiring veterinary support and ethical issues.^1^,^28^ VR models provide no physical interactions with real surgical tools, which is essential for instrument development; and it offers limited haptic feedbacks to the trainees, making the training scene unrealistic.^17^ Mechanical models play an important role in medical training due to the abstract anatomy demonstration, physical interactions, and durability.^4^,^14^ Organ phantom, such as the brain phantom,^7^ the cardiac phantom^10^,^23^ and the gastrointestinal (GI) phantom.^8^ have been developed for the surgical training and the testing of surgical robots. However, it still remains challenges to develop a realistic artificial organ phantom system that possesses detailed anatomy, soft material properties, realistic imaging results, and can offer quantitative feedback of the surgical outcome to the trainees. Recently, we combined advanced 3D printing and soft materials molding technologies to realize high-fidelity organ phantom for surgical simulation, including a GI interventional phantom,^11^ a soft kidney phantom^1^ and a resectable prostate phantom.^4^ These technical advances provide the possibilities to make realistic organ phantom using biomimetic soft materials.

Liver is an important organ in the digestive system. The hepatic diseases are regarded as the second leading cause of mortality amongst all digestive diseases in the US.^6^ The interventional procedures involving intrahepatic duct, including percutaneous transhepatic cholangiography (PTC), percutaneous transhepatic cholangiodrainage (PTCD), and endoscopic retrograde cholangio-pancreatography (ERCP), are becoming more and more popular. The estimated annual number of ERCPs is over 45,000 in the US with continually rising in the use of therapeutic ERCPs.^16^,^21^ The interventional procedures provide a minimally-invasive way to treat patients, with a shorter hospital stay and a lower surgical risk.^20^,^24^ However, the interventional procedures are difficult due to the following reasons: (1) high risk of complications, for example, the biliary infection, bile leaks, hemobilia, and pancreatitis; (2) high level of technical expertise and professional knowledge are needed to perform the interventional procedures; (3) few training opportunities available, which mostly existed in academic medical center or tertiary hospitals.^3^ As shown in the previous studies, a threshold of 180–200 ERCPs were recommended to achieve the competency.^9^ However, there are insufficient number of ERCP procedures for the fellowship training in most training centers.^18^ 74% GI fellows intended to perform ERCP even though they felt their ERCP training was inadequate.^15^ Therefore, the interventional procedure needs to be trained on a realistic liver phantom to meet the repetition threshold. Liver phantom has been made for surgical planning by visualizing important anatomical structures, such as the portal vein and the hepatic artery, as well as patient-specific tumors based on CT, magnetic resonance imaging (MRI), and cholangiography imaging data.^19^,^22^ However, these liver models do not provide hollow interior structures, such as the biliary system, and are mostly made of hard plastic materials.^13^,^19^,^22^,^29^ Dhir *et al.* reported an *ex vivo* liver model using 3D printed bile ducts and animal liver tissues, which is allowed for the endoscopic ultrasound bile duct drainage (EUS-BD),^5^ yet the anatomy structures of livers and bile ducts, as well as their corresponding position, are underrepresented. Tang *et al.* reported a liver model that was developed for choledochoscopic examination. The model was fabricated using 3D printing technology and then supported by VR for cholangioscopy examination.^25^ It shows a realistic anatomic structure of the biliary system, nevertheless, the outer shape of the liver is not suitable to perform transhepatic interventions or ultrasound-based procedures. To our knowledge, an anatomically-correct soft liver phantom with both hollow biliary system and the surrounding tissues has not been reported.

In this paper, we report a soft human liver phantom with detailed anatomical structures, including the interior biliary structures. The phantom uses soft material resembling liver tissues, which is validated using CT, ultrasound, and endoscopy imaging. It is feasible for the training of minimally-invasive procedures, e.g., ERCP and transhepatic interventions. By embedding electrical sensors in the phantom, the intervention can be evaluated quantitatively. The precision of the needle puncture into the biliary system under ultrasound guidance is assessed in real-time by the electrical resistance measurement and different sections of the biliary duct can be clearly distinguished. It offers a unique opportunity for interactive surgical training sessions and quantitative feedbacks to the trainees to efficiently improve their surgical skills, which are not possible before on existing organ models.

### Materials and Methods

## Design of the 3D Digital Model of the Liver

**Figure 1:**
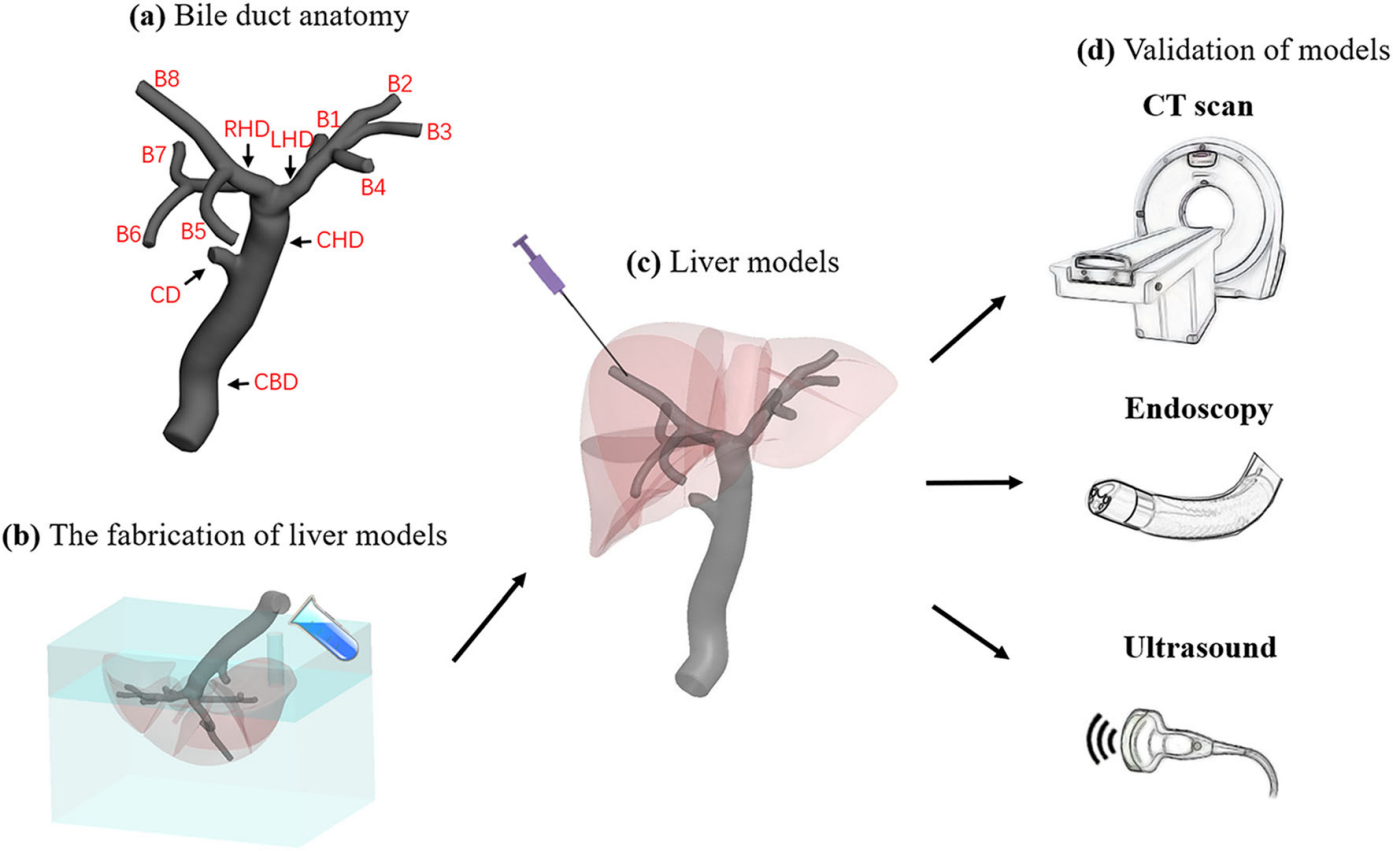
Schematics of the soft liver phantom with a biliary system for the training of medical procedures. (a) The anatomy of the bile duct, including: CBD, common bile duct; CD, cystic duct; CHD, common hepatic duct; LHD, left hepatic duct; RHD, right hepatic duct; B1-B8, segmental duct; (b) the phantom is fabricated by 3D printing and molding methods; (c) illustration of the liver model used in a transhepatic puncture procedure; (d) the model is validated by multi-modality medical imaging, including CT, endoscopy and ultrasound imaging.

Figure 1 shows the schematics of the soft liver phantom with a biliary system for the training of medical procedures. The 3D digital liver and biliary system models were built using the SolidWorks software (Dassault Systèmes SE, France). The 3D digital data of the liver outer shape was downloaded from an online database (BodyParts3D, https://lifesciencedb.jp/bp3d/), and it was scaled down to 80% in three dimensions using the Meshmixer software (Autodesk Meshmexer 3.5) in order to save 3D printing materials and time. The branches of intrahepatic bile ducts in different liver segments were designed according to medical experts’ knowledge and experience. The diameters of the bile ducts were set as the biliary dilation resembling clinical cases, i.e., 6 mm, 8 mm, 10 mm, 18 mm, 8 mm, 20 mm for the segmental ducts, left hepatic duct, right hepatic duct, common hepatic duct, cystic duct, and common bile duct, respectively, which represent the common pathologic intrahepatic biliary observations that need interventional treatments.^5^,^16^ Each segmental bile duct was distributed corresponding to the anatomy of the liver segments.

## Fabrication of the Liver Phantom

**Figure 2:**
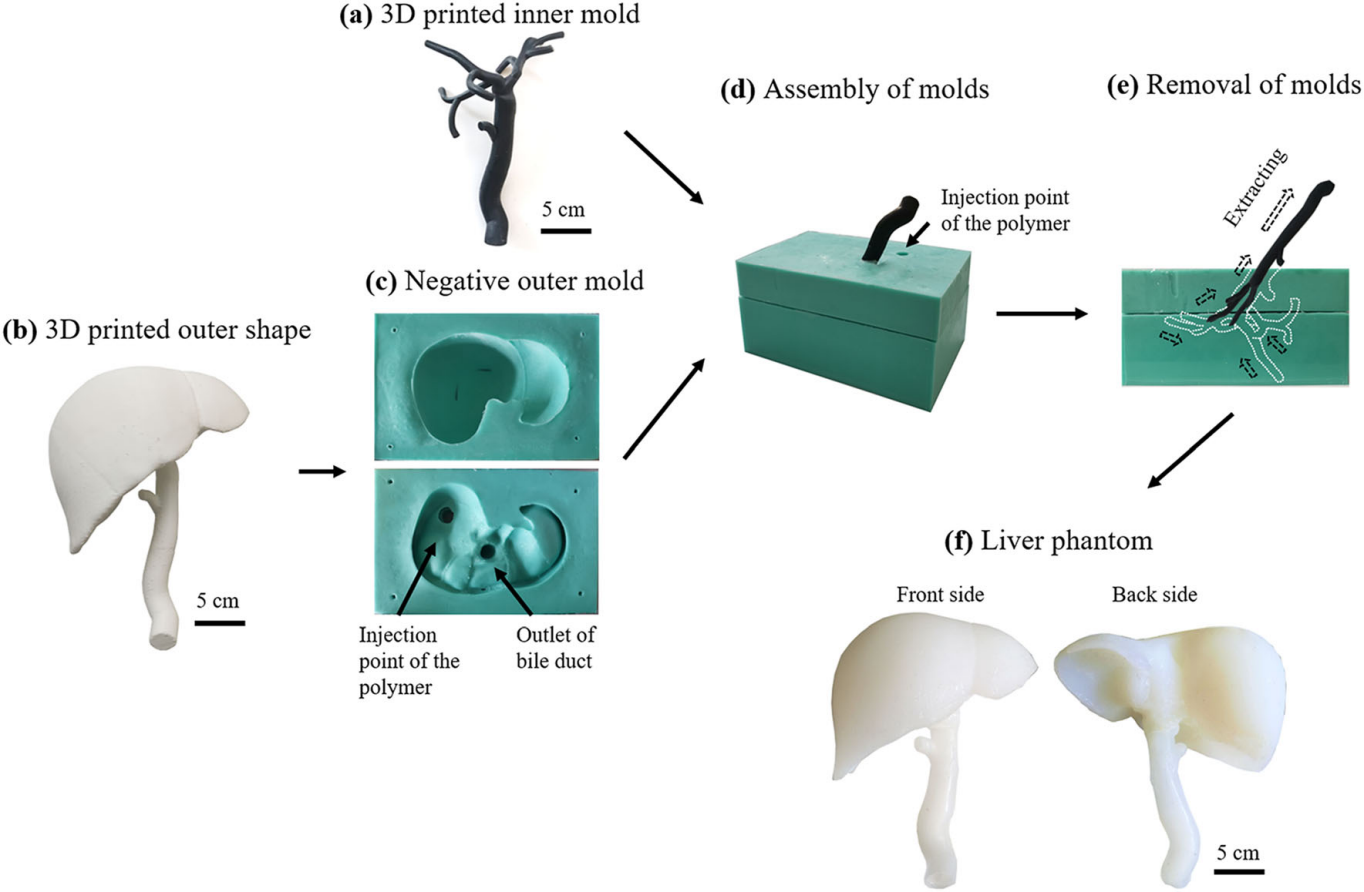
Workflow for the fabrication of the liver phantom. (a) The inner mold is 3D printed with a soft material; (b) the outer shape of the liver is 3D printed by a rigid material; (c) the negative outer mold; (d) the inner and outer molds are assembled and liquid polymer is poured into the mold; (e) the inner mold is extracted from the outer mold, and the phantom is demolded; (f) The obtained liver phantom.

Figure 2 shows the fabrication process of the liver phantom. The inner mold of the liver phantom, i.e., the biliary duct structure (of a bounding box size ~120 × 110 × 178 mm^3^) was printed using a rubber-like photopolymer Tangoblack+ material (Stratasys, Israel) on a 3D printer (Object 260 Connex, Stratasys) (Fig. 2a). And the outer shape of the liver (of a bounding box size ~167 × 127 × 124 mm^3^) was printed using acrylonitrile butadiene styrene (ABS) material (Fig. 2b) on a 3D printer (Stratasys Fortus 450mc, Stratasys). Silicone based material (Mold Star™, Smooth-On, PA, USA) was used to fabricate the negative outer molds based on the printed outer shape of the liver. Mold star was mixed with a volume ratio of 1:1 (Part A: Part B), and cured at 63 °C for 4 h, finally the outer shape of the printed model was mechanically removed. The inner mold was then assembled into the outer mold by fitting the position of extrahepatic ducts in the outer mold (Fig. 2d). Therefore, the inner mold was placed at the right position relative to the outer molds for molding. The outer molds were fixed with four positioning pins (3 mm × 3 mm ×10 mm). A silicone rubber material (Ecoflex 0020, Smooth-On, PA, USA) with micro glass beads (10 *μ*m average particle diameter, 1 wt%, Sigma-Aldrich, Germany) was thoroughly mixed mechanically and poured into the assembled mold. The mixture of the molding material was cured in an oven at 65 °C for 4 h. The inner mold was removed mechanically, and was extracted due to the high flexibility of the inner mold material (Fig. 2e). The extrahepatic bile duct was fabricated by the tip-coating method. The mold for the extrahepatic bile duct was immersed in a silicone rubber material (Dragon Skin™, Smooth-On) with the same micro glass beads, and then pulled out. A thin layer of silicone rubber was then cured overnight under room temperature. Finally, a liver model with the biliary tract were assembled using silicone adhesive with the extrahepatic duct (Fig. 2f).

## CT Imaging and Validation of the Phantom

**Figure 3:**
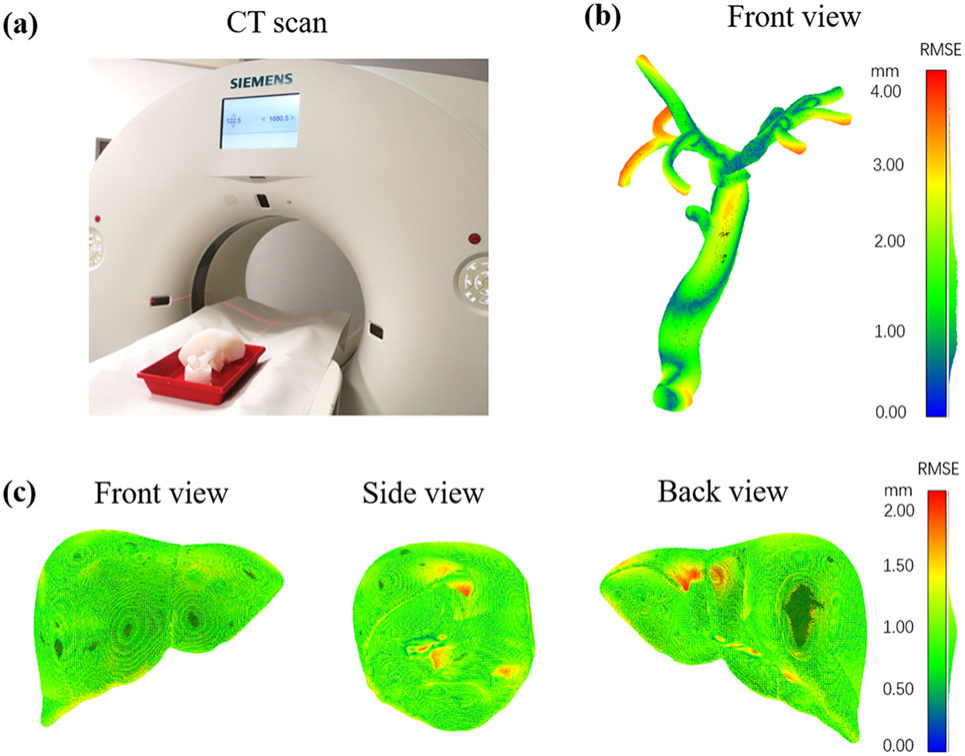
Evaluation of the accuracy of the liver phantom. (a) The phantom in a CT scanner; (b) quantitative error analysis of the biliary tract in the phantom compared to the digital model; (c) quantitative error analysis of the liver outer shape compared to the digital model. The surface colors represent the root mean square error (RMSE) in (b) and (c).

The liver phantom was scanned using CT (Somatom Force, Siemens, Germany), as shown in Fig. 3a. The data of the axial plane was obtained with a matrix size of 512 × 512, a field of view of 370 × 370 mm, and a slice thickness of 1 mm. The obtained IMA files were reconstructed in InVesalius v3.1.1 (Renato Archer Information Technology Center, Brazil). The shape of the biliary system and the liver outer shape were reconstructed separately. The threshold of the Hounsfield scale to reconstruct the biliary tract was set as the range between -1024 and -678 Hounsfield unit (HU), and the range for the liver was set between -478 and 1144 HU. The 3D reconstructed models were then compared with the 3D digital models of the liver and the biliary system for the quantitative analysis using a mesh editing software (CloudCompare v2.11). Two corresponding meshes in the STL files were aligned manually, and a cloud/cloud distance between the resulted model and the designed model was computed. The point-to-point distances were calculated. The spatial errors of the two models are displayed as pseudo color images in Figs. 3b and 3c. The RMSE of the distance between the liver phantom and the designed model was calculated to analyze the difference. RMSE refers to the square root of the distance difference between the resulted model and the designed model.

## Endoscopic Validation of the Phantom

**Figure 4:**
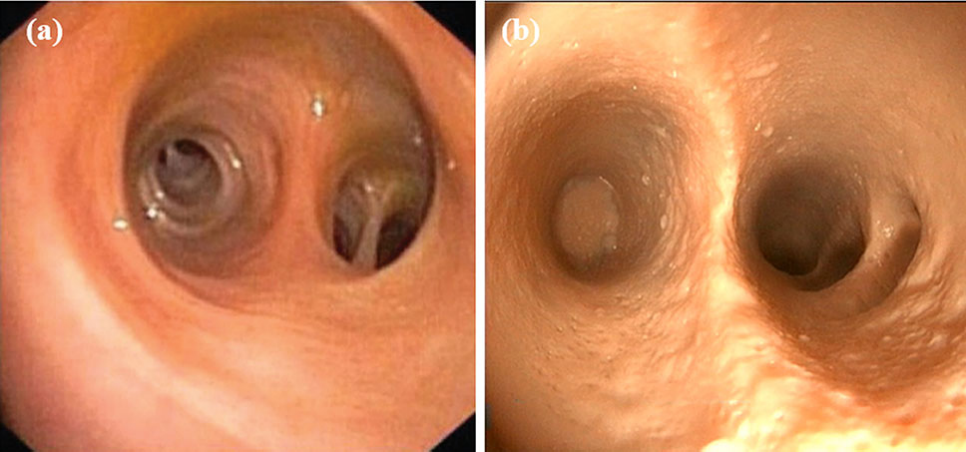
Validation of the liver phantom using endoscopy. (a) The view of the bile duct in a real human liver. The picture is taken from Ref. 23. (b) The view of the liver phantom.

A flexible endoscope was used for the endoscopic validation of the biliary duct of the phantom. A slim 16 French gastroscope (Slim video gastroscope, Silver Scope series, Karl Storz SE & Co. KG, Germany) was applied to visualize the inner parts of the biliary system in the liver phantom. The endoscope was connected to a camera control unit (Image1 S X-link, Karl Storz SE & Co. KG, Germany) and a light source (XENON 100 SCB, Karl Storz SE & Co. KG), and visualized by a HD monitor (9619 NB, Karl Storz SE & Co. KG). The snapshot of the endoscopic video is shown in Fig. 4b.

## Ultrasound Imaging and Validation of the Phantom

**Figure 5:**
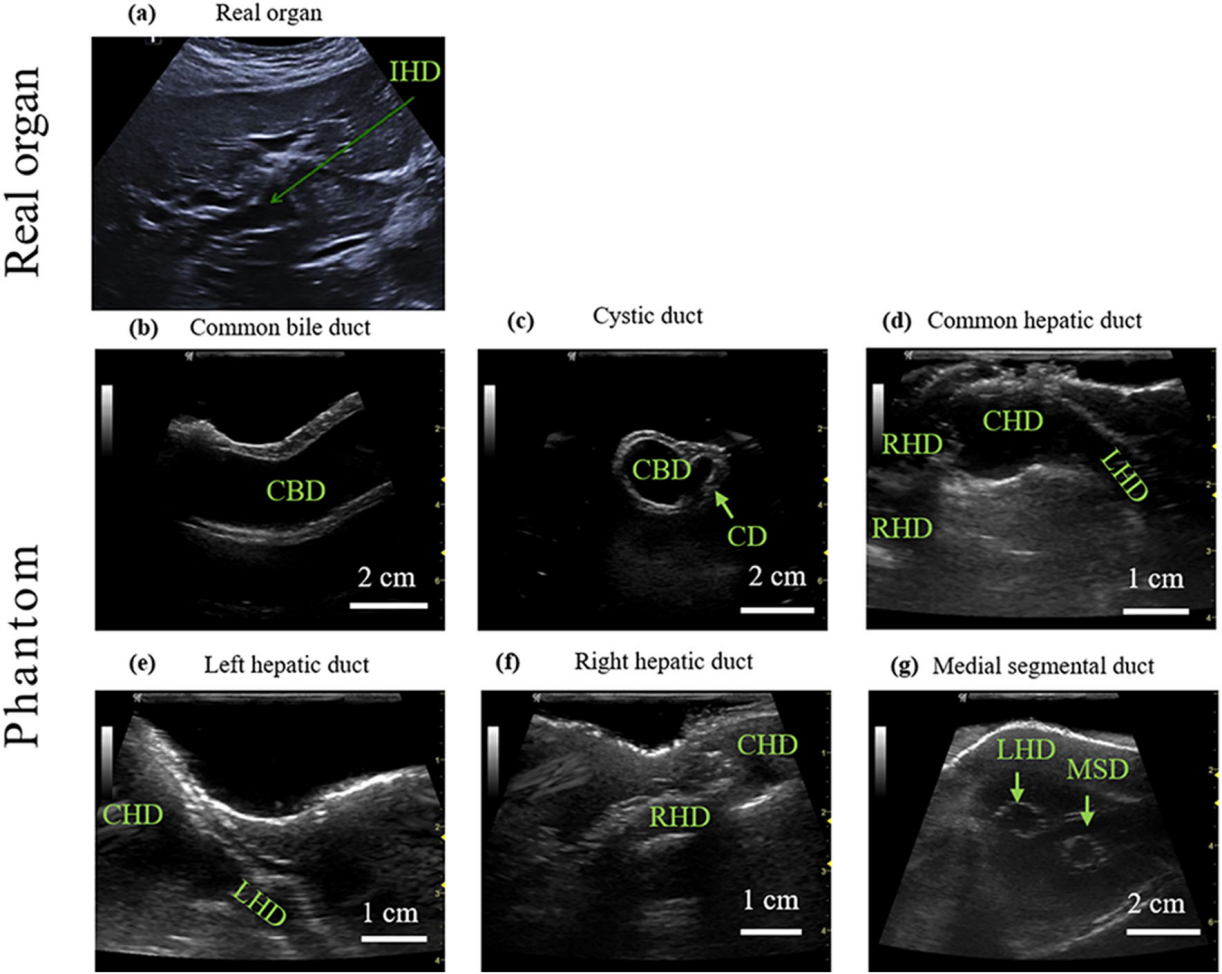
Ultrasonic images of the liver phantom compared to a real liver. (a) The ultrasonic image of a real liver organ, obtained from Ref. 21. (b)–(g) Ultrasonic images of the liver phantom. IHD, intrahepatic duct; CBD, common bile duct; CD, cystic duct; CHD, common hepatic duct; LHD, left hepatic duct; RHD, right hepatic duct; MSD, Medial segmental duct

The liver phantom was imaged using a clinical ultrasound imaging system (LOGIO P6, GE healthcare, Chicago, IL, USA) with a linear array ultrasonic transducer (10 MHz) in B-mode. The phantom was fixed under water and presented in the coronal plane. The important anatomical structures of bile ducts were visualized, including the common bile duct (CBD), cystic duct (CD), common hepatic duct (CHD), left hepatic duct (LHD) and segmental bile ducts. The ultrasound images of the bile ducts are shown in Fig. 5.

## Transhepatic Puncture Training on the Liver Phantom

The target biliary tract was visualized with the ultrasound imaging system using the same parameters mentioned above. Transhepatic puncture was performed using a 20-Gauge needle (Becton Dickinson S.A., Madrid, Spain). Interrupted needle insertion technique was used for the insertion of the needle until the tip of the needle was placed in the target biliary tract. The procedures were repeated for at least three times in each biliary tract.

## Assessment of the Needle Puncturing Procedure in the Intervention Simulation

**Figure 6:**
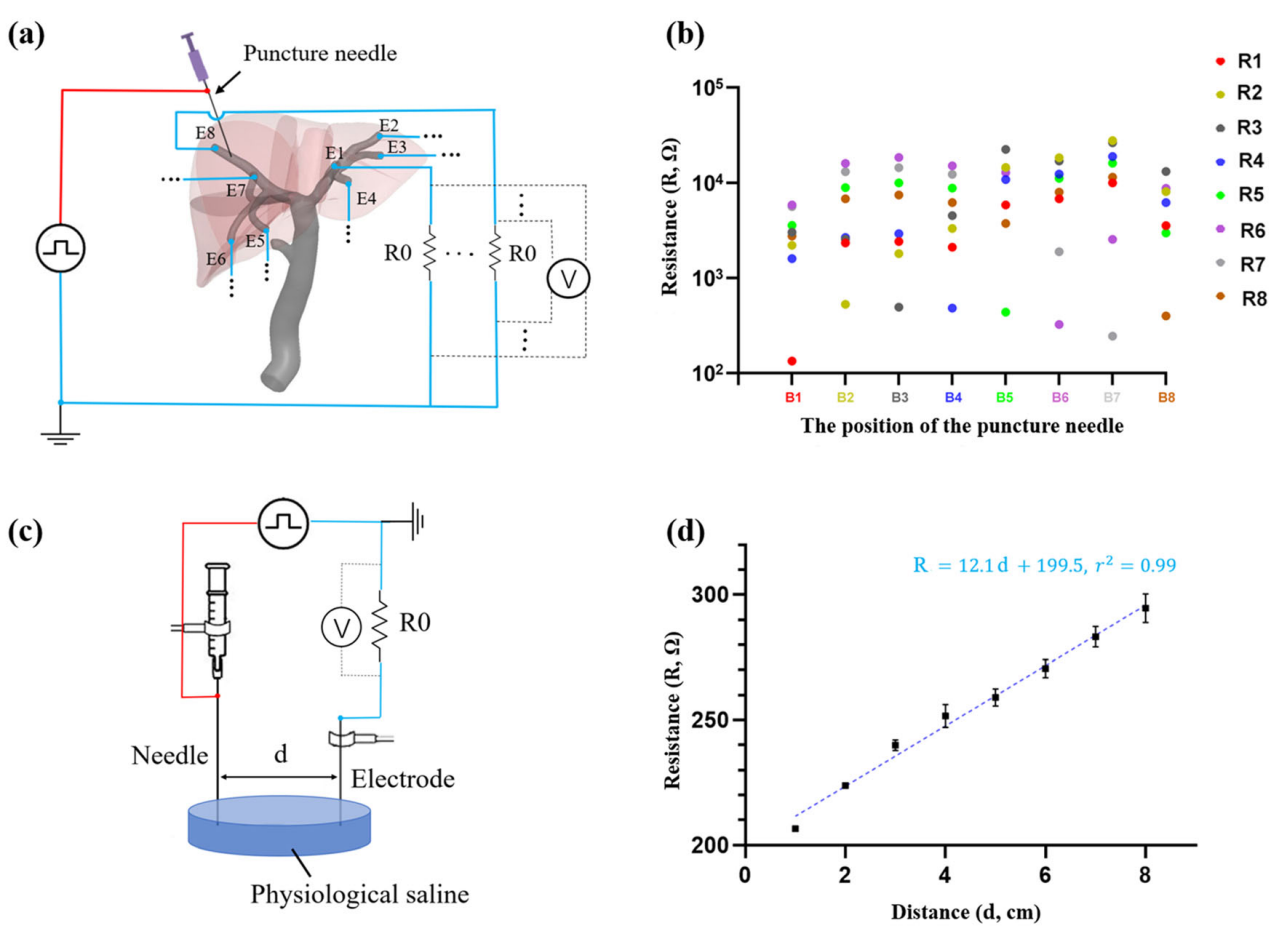
Electrical sensing of the transhepatic puncture procedure. (a) The schematic diagram. Eight electrodes were placed at the end of segmental biliary tract, filled with physiological saline solution and the resistance is measured respectively; (b) The resistances when placing the needle in different segmental biliary tracts. Each color represents an individual segment B1-B8, and the corresponding punctured segment shows the lowest resistance; (c) the schematic diagram of the set-up to measure the relationship between the distance and the resistance; (d) the resistance of the solution shows a linear relationship with the distance between the needle and the electrode.

A needle puncture tracking system was designed to assess whether the needle was successfully placed inside the target biliary tract. Figure 6a shows the schematic diagram of the puncture needle tracking system. Eight electrodes (E1 to E8, straight pin headers, RS Components GmbH, Germany) were placed at the tips of the corresponding biliary tracts (B1 to B8), respectively. The electrodes were interfaced to a laptop computer by a microcontroller (MEGA 2560, Arduino, Italy). The biliary tracts were fully filled with physiological saline (0.9% NaCl), simulating the bile juice. The saline acts as an electric conductor. After successfully performing a puncture procedure, the needle touches the solution and it closes the circuit in the biliary tract. Different amount of saline solution between the electrode and the needle acts as different resistors (R1 to R8). A square pulse (with an amplitude of 5.0 V and a period of 0.3 ms) was applied on the needle and the reference resistors R0 (470 Ω). The voltages on the reference resistors of the eight channels were measured to calculate the corresponding resistors of the saline solution (R1 to R8). The position of the puncture needle was determined by finding the lowest resistance, i.e., the shortest conductive path in the solution. The accuracy of the interventional procedure was also assessed by analyzing the resistances between the needle and a counter electrode as shown in Figure 6c.

### RESULTS

## CT Validation of the Phantom

Based on the CT scan, the digital model of the liver phantom was reconstructed and compared with the designed model to assess the accuracy of the fabrication process. The RMSE of the biliary system equals to 1.7 ± 0.7 mm. The bounding box dimension of the biliary tracts is approximately 147 mm (length) × 115 mm (width) × 243 mm (height). Thus, the mean error of the biliary tract model is around 1%. The largest error (corresponding to the red colors in Fig. 3b) is located mostly at the end of the biliary tracts due to its flexibility. Figure 3c shows the spatial error of the outer shape of the liver phantom, which exhibits an RMSE of 0.9 ± 0.2 mm. The bounding box dimension of the liver is approximately 209 mm (length) × 159 mm (width) × 157 mm (height). The mean error of the liver outer shape is lower than 0.7%. The red mark in Fig. 3c shows the largest error locates mainly on the visceral surface of the liver. These results indicate that the fabrication process accurately replicates the designed model with morphological details of both the liver and the biliary system. The precision of the fabrication process, including the mold preparation and the assembly processes, is high, which makes the liver phantom suitable for surgical training.

## Endoscopic Validation of the Phantom

An endoscopic assessment was performed to validate the possibility of cholangioscopy in the liver phantom. As shown in Fig. 4, the biliary tracts in the liver phantom truly resemble the typical characteristics of a human biliary system,^23^ including its morphology, color and surface texture. All biliary tracts can be intubated with the endoscope and clearly visualized. Each segmental duct in the phantom can also be examined in the cholangioscopy, the spatial orientation of the instrument is obtained as in a real organ.

Gastrointestinal surgeons were invited to perform endoscopic procedures on the liver phantom. Based on qualitative evaluation of the surgeons, the haptic feedback of the liver phantom during the endoscopy is similar to that of a real human liver, especially when the endoscope goes through the bifurcation of the left and right hepatic ducts. This is mainly due to the soft silicone material used in the phantom, which has an elastic modulus of ~60 kPa^1^ within the range of healthy human liver tissue of 0.5-70 kPa.^30^ When the endoscope was manipulated incorrectly and continuously further intubated, stronger resistance can be felt on the distal end of the endoscope. The haptic feedbacks offer realistic scenario as if the trainee is operating the endoscopy on a real patient.

## Ultrasound Validation of the Phantom

An ultrasonic image for the real organ is set as a comparison, which is obtained from Ref. 27. Compared to a real liver (Fig. 5a), the liver phantom closely resembles the acoustic signal of human tissues. The boundary of the liver, the tissue, and especially the shape of the biliary system was clearly visualized in the ultrasonic images. Both extra- and intrahepatic ducts are clearly recognized and traced in the liver phantom when performing an ultrasound examination. Figures 5b to 5g show the ultrasound images of different segments of the biliary tracts, including the common bile duct (CBD), cystic duct (CD), common hepatic duct (CHD), left hepatic duct (LHD), right hepatic duct (RHD), medial segmental duct (MSD). Other segmental bile ducts can be also detected in the phantom. The echogenicity of the liver parenchyma in the phantom exhibits realistic scattering effects due to the embedded micro glass beads in the silicone material. However, the bile duct that is over 4 cm underneath the surface cannot be clearly visualized due to the high acoustic attenuation of the silicone material.^2^ The silicone material has a speed of sound ranging from 993 to 1074 m/s, which is significantly lower than that of the human liver (around 1588 m/s).^2^ The material is chosen due to its durability, which is suitable for the needle puncture procedure. The fabrication method reported herein is general and can be extended to other materials, e.g., composite hydrogels that have similar acoustic properties to the human tissues.^4^

## Simulation of the Transhepatic Needle Puncture Procedure on the Phantom

The electric path in the solution is equivalent to a resistor and the position of the puncture needle can be localized in the biliary system by measuring the solution resistances at different electrodes. The value of each resistance corresponds to the distances between the needle and the electrode, and the linear dependence was mapped out in a calibration experiment (see below and Figs. 6c and 6d). The electric measurements were conducted with a short pulse of a period of 0.3 ms to avoid the effect of electrolysis of water. During the electrical measurement, the saline solution mainly acts as a resistor with a negligible phase delay. To calibrate the sensing system, the puncture needle was placed in the eight segmental biliary tracts (B1 to B8) respectively, and the resistances through all eight electrodes (R1 to R8) were recorded. The values of the resistances are shown in a log-scale in Fig. 6b. The lowest resistance is measured at the corresponding electrodes implanted in the same biliary duct, where the electrical path is the shortest, and the lowest resistance is almost one order of magnitude smaller than other values (Fig. 6b). For instance, when the puncture needle placed in the B1, the resistance to the closest electrode R1 is 134.9 ± 4.6 Ω, while the other resistances are in the range of 1600 Ω to 5600 Ω. Although the absolute resistances of the target resistors may differ due to the position of the needle and the needle geometry (e.g., the diameter, the shape of the tip), the difference of the values between the target resistor and other resistors are large enough to identify the correct location of the puncture needle by using a simple threshold method.

To evaluate the spatial resolution of the sensing method, a calibration experiment was carried out to draw the relationship between the resistance and the distance of two electrodes in solution (Fig. 6c). As shown in Fig. 6d, the resistance value of the saline solution increases linearly with the increase of the distance (the coefficient of determination *r*^2^ = 0.99). As the sensitivity of resistance sensing is around 0.1 Ω, the spatial resolution of the reported method is on the order of magnitude of 0.1 mm.

**Figure 7:**
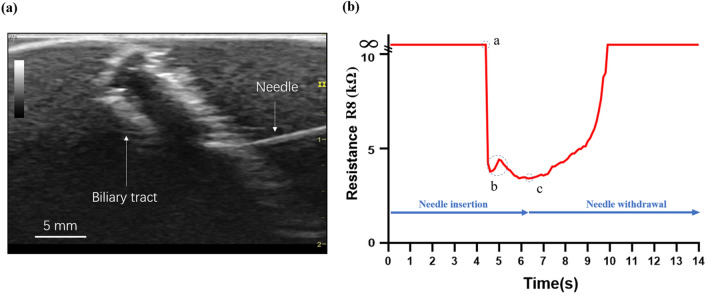
Electrical sensing of the dynamic transhepatic puncture process. (a) The needle puncture is performed under ultrasound guidance; (b) The change of the resistance of R8 during the procedure of B8 puncturing. Point a: the needle enters the biliary tract; Section b: the progressive motion of the needle; Point c: start the withdraw of the needle.

The transhepatic needle puncture procedure was performed on the liver phantom under the ultrasound imaging guidance. The right posterior superior duct (B8) was randomly set as a penetration target. An interrupted needle insertion technique was used, which is a commonly applied procedure in clinic. Specifically, the surgeon inserts the needle for ~2 mm and immediately withdraws it for ~1 mm and the progressive motion helps the surgeon sense the force feedback and avoid the perforation of important anatomical structures. Figure 7a shows the ultrasound image of the needle in the right posterior superior duct (B8). Figure 7b shows the record of the resistance R8 during the procedure of B8 puncturing (The recording of the ultrasound image and the resistance R8 are also shown in the Supplementary Video S1). The needle insertion phase corresponds to the section from Point a to Point c, and when the needle penetrated in the biliary tract, the value of R8 decreased rapidly. A peak is observed at Section b and the period of the peak is ~0.6 s, which is due to withdraw of the needle in the interrupted needle insertion technique. The withdraw phase occurred after Point c, when the needle was withdrawn from the biliary tract, the value of R8 increased gradually until infinity. Thus, the electric sensing method to localize the puncture needle shows high performance in both spatial and temporal resolution.

### DISCUSSION

High-fidelity surgical simulator plays an important role in the training performance and the acceptability of the simulation.^12^ The reported liver phantom presents highly accurate anatomical structures, especially with the hollow biliary system. It shows high-fidelity results in multi-modality medical imaging, including CT, ultrasound imaging, and endoscopy. As shown in the CT scan, the complex 3D shapes of both the liver and the biliary system were precisely replicated with the described approach. It is therefore possible to train different procedures at various locations, which is crucial in clinic practice, because the difficulties and strategies of interventional procedures highly depend on the position of the lesions. Apart from that, anatomic landmarks are also depicted in the phantom, for instance, gallbladder bed, the porta hepatis, gastric impression, *etc*. The anatomic landmarks are essential to recognize the liver anatomy and facilitate the surgical dissection. The liver phantom also provides realistic results for common clinical examinations, such as ultrasound and endoscopy, which enable the training of advanced medical procedures, such as ERCP, PTCD, and EUS-BD. Moreover, the mechanical and acoustic properties of the material are similar to the human liver tissue. Additional lesions made of different materials can also be placed to resemble diseased cases, such as cysts, hemangiomas, carcinoma. Further efforts will explore the effect of the medical training in advanced interventional and endoscopic procedures.

The fabrication method of 3D printing and molding offers an accurate and reproducible way to build soft organ models. Previously, the complex inner structure was fabricated using dissolvable inner molds,^1^,^22^ which are disposable, cost and labor intensive. The fabrication in the current study uses a demolding method that extracts the flexible inner mold from the liver outer shape, which saves 3D printing material, time and cost compared to the previous method.

The unique sensing system for surgical outcome evaluation is able to quantitatively analyze the performance of centesis, i.e., the accuracy of the puncture location. It provides real-time feedback to the trainee, sense new data that is difficult or impossible to be obtained in real organs and surgeries. The resistance curve in Fig. 7b shows the detailed maneuver of the interrupted insertion technique performed by the surgeon. This kind of needle insertion technique has a higher maximum insertion force and controllable movement that favors puncture and prevents complications.^7^ The small peak at Section b after the correct insertion of the needle in the biliary tract clearly reveals the withdraw step of the needle, which corresponds very well with the ultrasound imaging. It reveals the high temporal and spatial resolution of the proposed sensing method. The localization of the puncture needle is the most important parameter in the transhepatic puncturing procedure, as it directly correlates to the success and safety of the surgery. The electrical sensing method is general to localize an instrument in a phantom for the surgical training of minimally-invasive procedures. The training system assesses the puncturing accuracy and offers feedback to the physicians to learn the handling of sophisticated instrument and to optimize the surgical skills.

In conclusion, we report a soft human liver phantom with a hollow biliary system. The validations using CT, ultrasound imaging, and endoscopy show high resemblance to a human liver. Transhepatic needle puncturing procedure was successfully simulated and quantitatively evaluated on the phantom by an electrical sensing system. The liver phantom shows many potential applications including surgical simulation, endoscopic training and medical device testing.

## Supplementary Information

Below is the link to the electronic supplementary material.

Supplementary Video S1. The ultrasound video of the transhepatic puncture process and the plot of the electric resistance R8 during the procedure of B8 puncturing are shown in real time (MP4 5986 KB)

## Abbreviations

C: Celsius
CBD: Common bile duct
CD: Cystic duct
CHD: Common hepatic duct
CT: Computer tomography
ERCP: Endoscopic retrograde cholangio-pancreatography
EUS-BD: Endoscopic ultrasound bile duct drainage
Fig.: Figure
GI: Gastrointestinal
h: Hour(s)
HU: Hounsfield unit
kPa: Kilopascal
LHD: Left hepatic duct
mm: Millimeter
MRI: Magnetic resonance imaging
ms: Millisecond
MSD: Medial segmental duct
PTC: Percutaneous transhepatic cholangiography
PTCD: Percutaneous transhepatic cholangiodrainage
RHD: Right hepatic duct
RMSE: Root mean square error
USA: United States of America
VR: Virtual reality
*μ*m: Micron
Ω: Ohm
